# Different Human Immune Lineage Compositions Are Generated in Non-Conditioned NBSGW Mice Depending on HSPC Source

**DOI:** 10.3389/fimmu.2020.573406

**Published:** 2020-10-19

**Authors:** Nicholas J. Hess, Payton N. Lindner, Jessica Vazquez, Samuel Grindel, Amy W. Hudson, Aleksandar K. Stanic, Akihiro Ikeda, Peiman Hematti, Jenny E. Gumperz

**Affiliations:** ^1^ Department of Medical Microbiology and Immunology, University of Wisconsin-Madison School of Medicine and Public Health, Madison, WI, United States; ^2^ Department of Obstetrics and Gynecology, University of Wisconsin-Madison School of Medicine and Public Health, Madison, WI, United States; ^3^ Department of Medical Genetics, University of Wisconsin-Madison School of Medicine and Public Health, Madison, WI, United States; ^4^ Department of Microbiology and Immunology, Medical College of Wisconsin, Milwaukee, WI, United States; ^5^ Division of Hematology/Oncology, Department of Medicine, University of Wisconsin-Madison School of Medicine and Public Health, Madison, WI, United States

**Keywords:** human immune system mice, HSC, HPSC, c-KIT mutation, bone marrow, umbilical cord blood, human neutrophil engraftment, G-CSF mobilized peripheral blood

## Abstract

NBSGW mice are highly immunodeficient and carry a hypomorphic mutation in the *c-kit* gene, providing a host environment that supports robust human hematopoietic expansion without pre-conditioning. These mice thus provide a model to investigate human hematopoietic engraftment in the absence of conditioning-associated damage. We compared transplantation of human CD34^+^ HSPCs purified from three different sources: umbilical cord blood, adult bone marrow, and adult G-CSF mobilized peripheral blood. HSPCs from mobilized peripheral blood were significantly more efficient (as a function of starting HSPC dose) than either cord blood or bone marrow HSPCs at generating high levels of human chimerism in the murine blood and bone marrow by 12 weeks post-transplantation. While T cells do not develop in this model due to thymic atrophy, all three HSPC sources generated a human compartment that included B lymphocytic, myeloid, and granulocytic lineages. However, the proportions of these lineages varied significantly according to HSPC source. Mobilized blood HSPCs produced a strikingly higher proportion of granulocyte lineage cells (~35% as compared to ~5%), whereas bone marrow HSPC output was dominated by B lymphocytic cells, and cord blood HSPC output was enriched for myeloid lineages. Following transplantation, all three HSPC sources showed a shift in the CD34^+^ subset towards CD45RA^+^ progenitors along with a complete loss of the CD45RA^-^CD49f^+^ long-term HSC subpopulation, suggesting this model promotes mainly short-term HSC activity. Mice transplanted with cord blood HSPCs maintained a diversified human immune compartment for at least 36 weeks after the primary transplant, although mice given adult bone marrow HSPCs had lost diversity and contained only myeloid cells by this time point. Finally, to assess the impact of non-HSPCs on transplantation outcome, we also tested mice transplanted with total or T cell-depleted adult bone marrow mononuclear cells. Total bone marrow mononuclear cell transplants produced significantly lower human chimerism compared to purified HSPCs, and T-depletion rescued B cell levels but not other lineages. Together these results reveal marked differences in engraftment efficiency and lineage commitment according to HSPC source and suggest that T cells and other non-HSPC populations affect lineage output even in the absence of conditioning-associated inflammation.

## Introduction

Hematopoietic stem cell transplantation (HSCT) using autologous or allogeneic graft tissue sources is a potentially curative treatment for patients with malignant and non-malignant blood diseases. However, the clinical value of HSCT remains limited by its high potential for complications, including graft-*vs-*host-disease (GVHD), delayed immune reconstitution, and graft failure (1, 2). Recovery of neutrophil and platelet populations is a key indicator of engraftment success that usually occurs between 14 and 25 days post-transplant, with delays beyond this window increasing the chances of mortality due to infection (3, 4). Furthermore, the late reconstitution of other immune lineages, which can also demonstrate substantial variability, may carry long-term consequences for patient health, including influencing the likelihood of disease relapse (1–4). Factors that impact the capacity of human hematopoietic stem and progenitor cells (HSPCs) to engraft and differentiate, and the resulting dynamics of human hematopoietic reconstitution remain poorly understood, underscoring the need for new research strategies.

Xenogeneic models involving transplantation of human cells into animal hosts have the potential to shed new light on hematopoietic cell-intrinsic and -extrinsic factors that influence engraftment results and may thus provide an important path towards improving clinical outcomes (5–7). A key advance in this area was the development of immunodeficient mouse strains (*e.g.* NSG) that are highly permissive for engraftment of human hematopoietic cells because they lack adaptive lymphocytes due to a mutation that abrogates antigen receptor recombination (Prkdc^scid^) and are also deficient in innate lymphocytes due to the deletion of a key cytokine receptor gene (*γ*c or CD132) (6, 7). Further genetic modification of these strains has led to a variety of new options for modeling human hematopoietic engraftment. For example, mouse strains containing human knock-in gene replacements for key hematopoietic factors have recently been developed. These include NSG-SGM3 mice that have a triple knock-in of human stem cell factor (SCF), granulocyte–macrophage colony-stimulating-factor (GM-CSF) and interleukin-3 (IL-3), and MISTRG mice that contain human macrophage colony-stimulating-factor (M-CSF), GM-CSF, IL-3, thrombopoietin, and signal regulatory protein alpha (SIRPα) (8–10). Both strains have been shown to support high levels of multi-lineage human hematopoietic engraftment with superior representation of myeloid lineages and the MISTRG mouse also appears to sustain long-term hematopoietic progenitors (10). However, for efficient engraftment of human HSPCs in these strains it is necessary to administer pre-transplant conditioning (*e.g. γ*-irradiation).

Pre-transplant conditioning induces a variety of different inflammatory pathways and produces damage that may alter bone marrow microenvironment in ways that are still not fully understood (11). While pre-transplant conditioning is routinely used in clinical settings, there is substantial variation in the specifics of the regimens used, and the nature of the conditioning regimen is known to influence both the recovery of the immune compartment and the risk of GVHD (1–4). Myeloablative conditioning is associated with the fastest engraftment, though this is balanced by an increased probability of GVHD (1, 2, 12). Non-myeloablative and reduced intensity conditioning are less damaging and are associated with lower rates of GVHD, but show higher rates of delayed or failed engraftment (12). Thus, model systems that allow for analysis of transplantation outcomes in the absence of host conditioning may help to shed light on damage-independent factors that influence engraftment success. To this end, mouse strains containing mutations in the c-kit (SCF receptor or CD117) protein are particularly valuable, as they have been shown to support either murine or human HSPC engraftment in the absence of pre-conditioning (13–20).

An additional important factor influencing both hematopoietic recovery and GVHD is the source of tissue used for transplantation. The most common graft tissues used for clinical transplantation protocols are G-CSF mobilized peripheral blood (MB), bone marrow (BM), and umbilical cord blood (CB), in that order (21, 22). While the incidence of GVHD and times to immune recovery vary according to the source of graft tissue (3, 4), it remains unclear how these outcomes are impacted by intrinsic differences in the HSPCs found in different types of human graft tissue or by non-HSPC populations that are co-transplanted as part of these grafts. In this study, we sought to compare engraftment outcomes resulting from transplantation of human HSPCs isolated from BM, MB, or CB and to assess the impact of lineage-committed cells on hematopoietic reconstitution. Since the inflammatory status of the host environment may significantly affect the functions of both HSPCs and differentiated cells, to avoid conditioning-associated damage we used NBSGW mice, in which a hypomorphic mutation in the *c-kit* gene (Kit^W41J^) has been crossed into the highly immunodeficient NSG (NOD.Cg-Prkdc^scid^Il2rg^tm1Wjl^) strain (13–20). Remarkably, while prior studies have demonstrated improved human myeloid engraftment in these or related *c-kit* mutated mice (13–20), the comparison performed here demonstrates that the NBSGW strain supports marked differences in human granulocytic, B lymphocytic, and myeloid lineage output depending on the HSPC source and points to an unexpected impact on B cell engraftment levels when T lymphocytes are present within graft tissue.

## Methods

### Isolation of Primary Human HSPCs

All work involving human cells was performed under UW Minimal Risk IRB protocols 2018-0304 (JG), 2016-0298 (PH), and 2017-0870 (JG). Human BM and MB tssues were collected from the remnants of left-over bags and filters used for clinical HSCT procedures at the University of Wisconsin-Madison. CB samples were acquired either from the University of Colorado’s ClinImmune Labs cord blood bank or the Medical College of Wisconsin’s tissue bank. Mononuclear cells were isolated by ficoll density-gradient centrifugation (1,100×g for 15 min with 0 brake) to remove red blood cells (RBCs), neutrophils, and other non-leukocytes. Where indicated, T cells were depleted using StemCell Technologies RosetteSep T cell-depletion kit (catalog #15661), and HSPCs were isolated using StemCell Technologies EasySep Human CD34 positive selection kit II (catalog #17856), which first depletes lineage positive cells, then positively selects CD34^+^ cells. The resulting cell population was typically >90% CD34^+^, with the remaining cells all lineage negative. Prior to transplantation, cells were counted, washed, and resuspended in phosphate buffered saline (PBS).

### Transplantation Into NBSGW Mice

All animal work was completed in accordance with UW-Madison IACUC protocol M005199. NBSGW immunodeficient mice (NOD.CG-Kit^W-41J^Tyr^+^Prkdc^scid^IL2rg^tm1Wjl^/ThomJ) were initially purchased from Jackson Laboratories, then bred and maintained in a specific pathogen free facility using aseptic housing. Experiments were performed using both male and female mice (equal numbers of each sex); results are not stratified according to sex, since no significant differences in results were observed between the sexes. All mice were between the ages of 6–10 weeks at the time of transplantation. Human cells suspended in a volume of 150 µl of PBS were injected retro-orbitally while the mice were under isoflurane anesthesia. Blood draws were performed at indicated time points by retro-orbital bleeding, using heparin coated capillary tubes with the mouse under isoflurane anesthesia. At the experiment endpoint, mice were euthanized by CO_2,_ and tissues collected for analysis. Bone marrow was prepared by flushing tissue from the inside of the femur and tibia of the left leg. Mice were monitored for signs of GVHD (weight loss ≥5% of maximum body weight) by weekly weighing as described previously (23). Mice characterized as experiencing GVHD were removed from the analysis.

For analysis of engraftment following secondary transplantation, primary transplants were first performed on four mice using a dose of 2.5E^6^ purified HSPCs. After 12 weeks, bone marrow (both femurs) from these four mice was pooled and purified by density gradient centrifugation (Ficoll) to remove non-leukocytes. Four naïve mice were then transplanted with 3E^7^ pooled bone marrow cells. Flow cytometric analysis of the pooled MNC used for the secondary transplant showed that ~2.5E^6^ HSPC of the injected cells were human cells expressing CD34.

### Flow Cytometry

Singe cell suspensions were blocked in PBS with 10% human serum and stained for flow cytometry using fluorophore-conjugated antibodies purchased from BioLegend: CD3 (OKT3), CD10 (HI10A), CD19 (HIB19), CD33 (HIM3-4), CD34 (8G12), CD38 (HIT2), mCD45.1 (A20), CD45RA (HI100), CD56 (HCD56), CD66b (610F5), CD123 (6H6), and PanHLA (w6/32). All samples were analyzed on a BD LSRII flow cytometer equipped with three lasers (nine filter channels), quantified using Precision Count Beads (BioLegend) and analyzed using FlowJo V10.5.

### Immunofluorescence of Murine Bone Tissue

Following CO_2_ euthanasia, mice were immediately perfused with PBS, followed by 4% paraformaldehyde (PFA). Femurs were removed and immersion fixed in 4% paraformaldehyde (PFA) for 24 h at 4°C. Femurs were then decalcified in 20% EDTA for 4 days at room temperature. Femurs were rinsed, dehydrated, and embedded in paraffin. Paraffin blocks were sectioned 5 µm thick and mounted on glass slides. Sections were de-paraffinized, rehydrated to PBS, and heated in citric acid buffer (10 mM sodium citrate, 0.05% tween-20, pH 6.0) for antigen retrieval. Sections were blocked in PBS with 0.5% Triton X-100 and 2% normal donkey serum for 1 h at room temperature. Sections were then incubated at 4°C overnight with Leukocyte Common Antigen (LCA) Cocktail (Biocare Medical, Pacheco, CA) diluted at 1:100 in the diluent solution supplied by the manufacturer. Sections were rinsed in PBS and incubated in 1:400 diluted secondary antibody (Cy3-conjugated anti mouse IgG, Jackson ImmunoResearch Laboratories, West Grove, PA) in block solution for 1 h at room temperature. Following PBS rinse, sections were stained with 4′,6-diamidino-2-phenylindole (DAPI). Slides were imaged using a Nikon A1R+ confocal microscope (Nikon, Tokyo, Japan).

### Statistics

**Figure 1:**
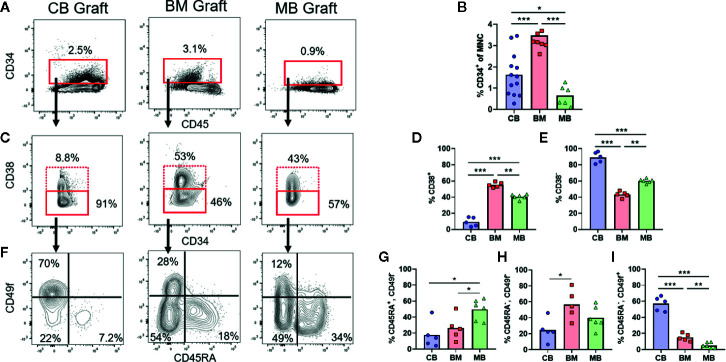
Composition of human HSPC populations varies among graft sources. Flow cytometric analysis of HSPC populations in primary graft tissues. **(A)** Representative plots of panHLA-class-I^+^ human cells from umbilical cord blood (CB), bone marrow (BM), and G-CSF mobilized peripheral blood (MB), showing the percentage of CD34^+^ cells (red box) in each graft. **(B)** Aggregated results showing percentage of CD34^+^ cells detected in individual graft samples. Symbols show results from individual samples of CB (blue), BM (red), or MB (green) tissue, with bars representing the arithmetic means. **(C)** Representative plots showing flow cytometric staining of CD34^+^ cells for CD38. **(D, E)** Aggregated results showing the percentage of cells in the CD38^+^ **(D)** or CD38^−^ **(E)** gates. **(F)** Representative plots showing flow cytometric staining of CD34^+^CD38^−^ cells for CD45RA and CD49f. **(G–I)** Aggregated results showing the percentage of CD34^+^CD38^−^ cells corresponding to uncommitted progenitors (CD45RA^+^CD49f^−^, **G**), multipotent progenitors (CD45RA^−^CD49f^−^, **H**), and long-term HSCs (CD45RA^−^CD49f^+^, **I**). *p < 0.05; **p < 0.01; ***p < 0.001.

Replicate mice were transplanted in each experiment using the indicated graft sources and doses, and results from like conditions of independent experiments were aggregated for statistical analyses. Data were analyzed for significance using a two-tailed, unpaired, non-parametric t-test (Mann–Whitney analysis). Symbols on plots represent results from individual mice (or individual graft samples in **Figure 1**), and bars or lines show the means. Data sets from analyses of cell number are plotted using a logarithmic scale with geometric means shown, while analyses of cell frequencies (percentages) are plotted using a linear scale with bars or lines showing the arithmetic means.

## Results

### Characterization of Human HSPCs From Different Graft Sources

The three commonly used HSCT graft tissues (CB, BM, and MB) all contain CD34^+^ HSPCs capable of re-populating hosts but are collected from different physiological niches (umbilical cord/placental blood, bone marrow interstitial space, and peripheral blood, respectively). Additionally, donors of MB grafts undergo pre-treatment with G-CSF, which may be associated with changes to HSPC function in addition to inducing migration of HSPCs from the bone marrow into the blood, while CB grafts are from less developmentally mature donors and are exposed to factors associated with pregnancy (24–26). Therefore, HSPCs in these three graft tissues are expected to differ in multiple ways. We performed flow cytometric analysis to gain an initial assessment of HSPCs from these three tissue types. Similar to previous findings, our analysis revealed that on average the percent of CD34^+^ cells was lowest in MB graft tissue, while BM had the highest average, and cord blood samples were highly variable yielding an intermediate average (**Figures 1A, B**) (23). We performed flow cytometric analysis using a previously published gating strategy to further identify the CD38^+^ committed progenitors and CD38^−^ uncommitted progenitor populations within the CD34^+^ pool (**Figures 1C–E**) (27, 28). We observed that CB grafts had the highest percentage of uncommitted progenitors followed by MB and BM, respectively (**Figures 1D, E**). We further divided the CD38^−^ population into long-term HSC (CD45RA^−^, CD49f^+^), MPP (CD45RA^−^, CD49f^−^) and an uncommitted progenitor (CD45RA^+^, CD49f^−^) population. From this analysis, CB grafts had the highest percentage of long-term HSCs, while the MPP population was dominant in BM, and MB showed enrichment for the uncommitted progenitor population (**Figures 1F–I**). These results are consistent with clinical data showing that a lower absolute number of CB-CD34^+^ cells is required for engraftment, but hematopoietic recovery takes longer than for grafts of other tissue sources, presumably because CB grafts contain fewer differentiated progenitors (3, 4, 29, 30).

### Human Cell Chimerism Is Established in Murine Bone Marrow and Peripheral Tissues

**Figure 2:**
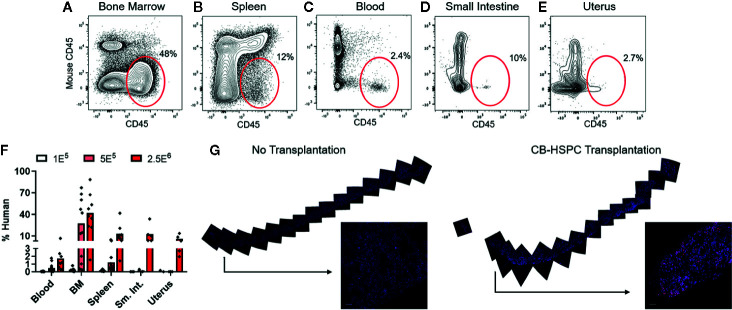
Transplantation of HSPCs is associated with colonization of bone marrow and peripheral tissues by human cells. Bone marrow and peripheral tissues (spleen, blood, small intestine, and uterus) were collected at 12 weeks post-transplantation from mice given varying doses of BM-HSPCs (1E^5^, 5E^5^, 2.5E^6^). **(A–E)** Representative plots showing flow cytometric staining of human cells (red oval gates) in a mouse transplanted with 2.5E^6^ BM-HSPCs. **(F)** Aggregated data showing percentages of human cells within the total (human CD45^+^ + murine CD45^+^) hematopoietic compartment from the indicated tissues of mice engrafted with the indicated BM-HSPC doses. Symbols show results from individual mice, bars represent the arithmetic means. **(G)** Cells expressing human CD45 were detected by immunofluorescence of whole bone tissue collected at 12 weeks post-transplant from a mouse given 2.5E^6^ CB-HSPCs (right side), compared to a negative control unengrafted mouse (left side). Staining of human CD45 is shown in red; blue color shows staining of cell nuclei by DAPI.

To confirm successful engraftment of human immune cells in this model, we performed flow cytometric analyses to test them in bone marrow and peripheral tissues. Human cells were detectable at 12 weeks post-engraftment in the bone marrow and also in all other murine tissues tested, including spleen, blood, small intestine, and uterus (**Figures 2A–F**). The abundance of human cells at these sites appeared dependent on the dose of HSPCs used for engraftment (**Figure 2F**). The bone marrow typically showed the highest levels of human chimerism as a percentage of the total cells within the murine tissue (**Figure 2F**). To further characterize the bone marrow engraftment, we performed immunofluorescence staining on sections of whole femur (**Figure 2G**). Femur of an engrafted mouse showed abundant cells staining positively for human CD45 that were distributed throughout the bone and appeared most concentrated near endosteal areas (**Figure 2G**, right side), and analysis of similarly prepared femur from an unengrafted mouse indicated that non-specifically stained cells were rare (**Figure 2G**, left side). Together, these results strongly support that non-conditioned NBSGW mice are productively engrafted following transplantation of purified human HSPCs.

### Efficiency of Engraftment According to HSPC Source

**Figure 3:**
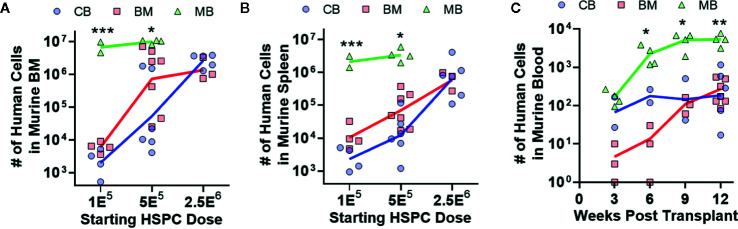
Human hematopoietic output is dependent on HSPC dose. The relative abundance and kinetics of human HSPC expansion in NBSGW mice was determined for each graft source. **(A)** Total number of human cells in the bone marrow collected from one leg (femur + tibia) at 12 weeks post-transplantation according to the starting HSPC dose. Symbols show individual mice that received HSPCs isolated from CB (blue), BM (red), or MB (green), with lines indicating geometric means. **(B)** Total number of human cells in the murine spleens at 12 weeks post-transplantation. **(C)** Total number of human cells in 100 μl of murine peripheral blood, collected at the indicated times post-transplantation. Results are from transplantation of 5E^5^ HSPCs. *p < 0.05; **p < 0.01; ***p < 0.001.

To investigate hematopoietic output of HSPCs from the three different graft sources, we transplanted titrated doses of lineage-negative CD34^+^ cells from each type of graft tissue into NBSGW mice, and after 12 weeks we assessed the total number of human cells in one femur (**Figure 3A**) or in the spleen (**Figure 3B**). Dose response curves appeared similar for murine bone marrow and spleen transplanted with CB- and BM-HSPCs (**Figures 3A, B**). Remarkably, MB-HSPCs generated one to three orders of magnitude more total human cells in these murine tissues as a function of starting HSPC dose (**Figures 3A, B**). We also assessed the number of human cells present in 100 μl murine blood, at 3 week intervals following transplantation (**Figure 3C**). This analysis demonstrated that mice transplanted with MB-HSPCs reached significantly higher levels of human chimerism within 9 weeks. Thus, compared to CB- or BM-HSPCs, MB-HSPCs engrafted more efficiently at low doses and also produced more rapid peripheral reconstitution in this model.

### HSPC Source Influences Lineage Output

**Figure 4:**
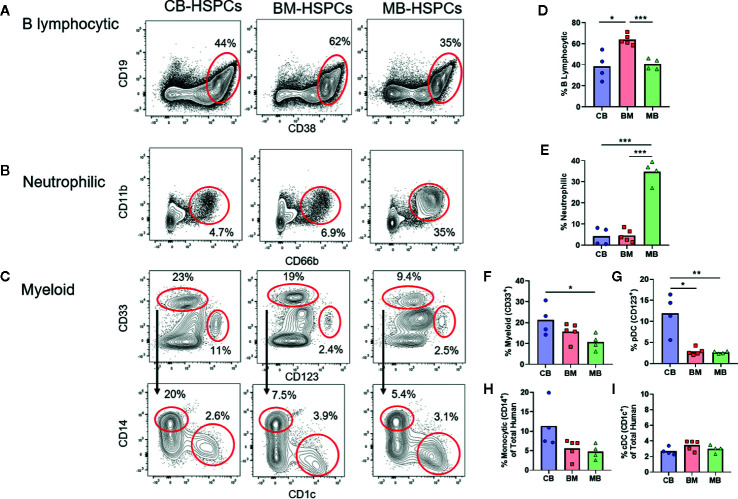
Source of human HSPCs influences hematopoietic output. The lineage output resulting from transplantation of CB-, BM-, or MB-HSPCs was examined by flow cytometry of murine bone marrow after 12 weeks. **(A–C)** Representative plots showing flow cytometric analysis of human cells (panHLA-I^+^, murine CD45^−^) in the bone marrow of NBSGW mice given 5E^5^ HSPCs. **(A)** Identification of B lymphocytic cells (CD19^+^, CD38^high^). **(B)** Identification of neutrophilic cells (CD11b^+^, CD66b^+^). **(C)** Identification of myeloid lineage cells (CD33^+^ gate in top row), that are comprised of monocytic cells (CD14^+^ gate in bottom row) and DCs (CD1c^+^ gate in bottom row). Additionally, a progenitor population that is thought to give rise to plasmacytoid DCs and other cells is present (CD33^−^CD123^+^ gate in top row). **(D–I)** Plots showing the percent of each population within the human compartment of mice given the indicated sources of HSPCs. Symbols show results from individual mice, bars represent the arithmetic means. *p < 0.05; **p < 0.01; ***p < 0.001.

We next characterized the composition of the human compartments generated within mice transplanted with the three sources of HSPCs. Flow cytometric analysis of the murine bone marrow at 12 weeks after transplantation showed a heterogeneous population of human cells that we further identified as B lymphocytic [CD19^+^, CD38^high^, CD10^+^ (not shown)], neutrophilic (CD11b^+^, CD66b^+^), myeloid cells consisting of monocytic (CD33^+^, CD14^+^) and dendritic (CD33^+^, CD1c^+^) cells, and a progenitor population that was low/intermediate for CD33 and positive for CD123 (**Figures 4A–C**). T cells (*i.e.* CD3^+^) were not detected following transplantation of any of the types of HSPCs, which is mostly likely a consequence of thymic atrophy in these mice.

Strikingly, the composition of the human compartment in the murine bone marrow at 12 weeks showed marked differences according to the source of HSPC transplanted. The human immune compartment of mice transplanted with BM-HSPCs was dominated by B lineage cells (~60%), whereas B lymphocytic cells comprised only about 30–40% of the human immune compartment of mice transplanted with CB- and MB-HSPCs (**Figure 4D**). Mice transplanted with MB-HSPCs showed a dramatic enrichment for neutrophilic lineage cells (~35%), whereas neutrophilic cells comprised only about 5% of the human compartment in mice that received BM- or CB-HSPCs (**Figure 4E**). Finally, mice that received CB-HSPCs showed an approximate twofold and threefold enrichment for CD33^+^ and CD123^+^ lineages respectively compared to mice transplanted with BM- or MB-HSPCs (**Figures 4F, G**). The percentage of monocytic cells ranged from about 5% in mice that received BM- or MB-HSPCs to about 10% in mice transplanted with CB-HSPCs (**Figure 4H**), while the percentage of CD1c^+^ DCs (~3%) did not show significant differences according to HSPC source (**Figure 4I**).

### HSPC Population Shifts Towards Progenitor Phenotypes Following Transplantation

**Figure 5:**
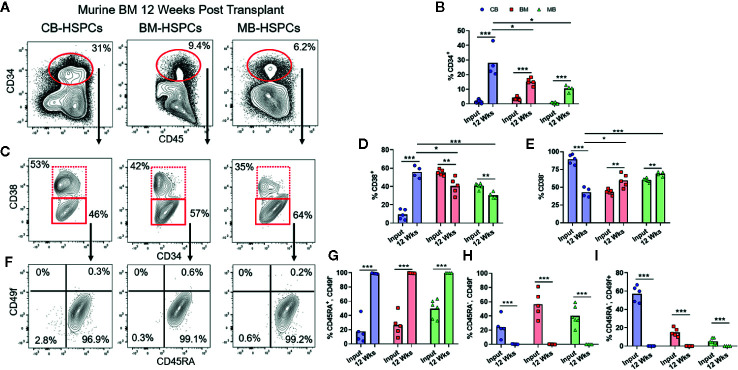
Shift towards short-term HSPC phenotype after transplantation. Flow cytometric analysis was performed to assess the composition of the CD34^+^ HSPC population within the human compartment in the murine bone marrow at 12 weeks post-transplantation. **(A)** Representative flow cytometry plots showing identification of human CD34^+^ population. **(B)** Plot showing percentage of CD34^+^ cells detected in bone marrow of mice transplanted with the indicated sources of HSPCs compared to the frequency of total CD34^+^ cells in the starting graft tissue (Input). **(C)** Representative plots showing flow cytometric staining of human CD34^+^ cells in murine bone marrow for CD38. **(D, E)** Percentage of cells in the CD38^+^ **(D)** or CD38^−^ **(E)** gates after transplantation compared to the starting graft (Input). **(F)** Representative plots showing flow cytometric staining of human CD34^+^CD38^−^ cells in the murine bone marrow for CD45RA and CD49f. **(G–I)** Percentage of CD34^+^CD38^−^ cells corresponding to uncommitted progenitor (CD45RA^+^CD49f^−^, **G**), multipotent progenitor (CD45RA^−^CD49f^−^, **H**), and long-term HSC (CD45RA^-^CD49f^+^, **I**) gates after transplantation compared to the starting graft (Input). Symbols show results from individual mice; bars represent the arithmetic means. *p < 0.05; **p < 0.01; ***p < 0.001.

We next evaluated the characteristics of the CD34^+^ HSPC population in the bone marrow of transplanted mice (**Figure 5A**). Regardless of the source of HSPCs, the percentage of human cells expressing CD34 was significantly expanded at 12 weeks post-transplantation compared to the starting graft tissue (**Figure 5B**). We performed the same sequential gating strategy used to characterize the CD34^+^ subset of the starting grafts (**Figure 1**). Compared to the respective starting grafts, the percentage of committed progenitors (CD38^+^) was markedly increased for CB-, but somewhat decreased after transplantation of BM- and MB-HSPCs (**Figures 5C–E**). Strikingly, for all HSPC sources, the CD38^−^ population showed a dramatic shift towards uncommitted progenitors (CD45^+^CD49f^−^) and away from MPPs (CD45^−^CD49f^−^) and long-term HSCs (CD45^−^CD49f^+^) (**Figures 5F–I**). Indeed, we did not detect any human cells bearing a long-term HSC phenotype in the bone marrow from any of the mice analyzed, and even cells bearing an MPP phenotype appeared extremely rare (**Figures 5F–I**).

**Figure 6:**
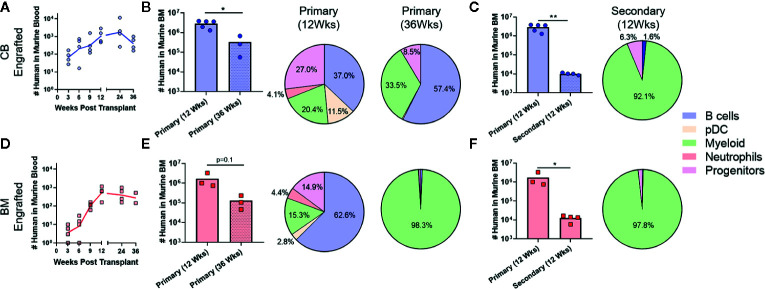
Transplantation of CB-HSPCs produces a longer duration of multi-lineage engraftment than BM-HSPCs. Analyses were performed to evaluate the duration of the multi-lineage expansion produced by the initial HSPC transplantation as well as the ability of engrafted HSPC to re-populate a naïve host. **(A)** Blood draws were performed every 3 weeks on NBSGW given 2.5E^6^ CB-HSPCs until week 12 after which they were performed every 12 weeks until 36 weeks. **(B)** Bar graph showing the total number of human cells in the bone marrow collected from one leg (femur + tibia) as determined by flow cytometry in mice 12 or 36 weeks post-transplant. The lineage composition of the human cells is shown as a pie graph. **(C)** Bone marrow collected from four mice initially transplanted with 2.5E^6^ CB-HSPCs was combined and subjected to density gradient centrifugation, then 3E^7^ mononuclear cells was transplanted into each of four naïve NBSGW mice. The number of transplanted HSPCs was approximately 2.5E^6^/mouse. The plot shows total number of human cells in the bone marrow collected from one leg (femur + tibia) at 12 weeks after primary *vs.* secondary transplant. Pie chart shows lineage composition at 12 weeks after secondary transplant. **(D–F)** Results from similar analysis using BM-HSPCs. Symbols show results from individual mice, and lines or bars show the geometric means. *p < 0.05; **p < 0.01.

Since prior studies have established that the transition from HSC to MPP to uncommitted progenitors occurs as a step-wise process, with each population capable of repopulating itself as well as giving rise to more committed populations (27, 28), the absence of HSC and MPP populations in the murine bone marrow after 12 weeks suggested that the NBSGW host promotes short-term hematopoietic activity with little stem cell self-renewal. To investigate further, we evaluated the duration of engraftment in mice transplanted with CB- or BM-HSPCs. In both cases, human chimerism was maintained in peripheral blood for at least 36 weeks post-transplantation (**Figures 6A, D**), but the total number of human cells in the bone marrow declined by about 10-fold (**Figures 6B, E**). In mice transplanted with CB-HSPCs, the human compartment in the murine bone marrow shifted over time towards increased representation of B lymphocytes and myeloid lineages and reduced frequency of progenitors (**Figure 6B**). In contrast, mice transplanted with BM-HSPCs underwent a dramatic loss of lineage diversity and by 36 weeks post-transplantation the human compartment in the murine bone marrow was comprised almost exclusively of CD33^+^ myeloid cells (**Figure 6E**). To directly evaluate the capacity for self renewal, we performed a serial transfer of murine bone marrow from mice transplanted with CB- or BM-HSPCs into naïve NBSGW mice. Bone marrow was collected at 12 weeks post-transplantation from four mice given CB- or BM-HSPCs, pooled and subjected to density gradient centrifugation to isolate mononuclear cells, then split into equal doses and transplanted into four naive mice. Murine bone marrow was harvested for analysis at 12 weeks after the secondary transplant. For mice given either CB- or BM-HSPCs, the number of human cells in the murine bone marrow was about 2 logs lower than the primary transplant, and the human compartment was almost exclusively comprised of CD33^+^ myeloid cells (**Figures 6C, F**). Together, these results suggest the NBSGW strain promotes short-term hematopoietic activity of transplanted human HSPCs.

### Impact of Non-HSPCs in Graft Tissue

**Figure 7:**
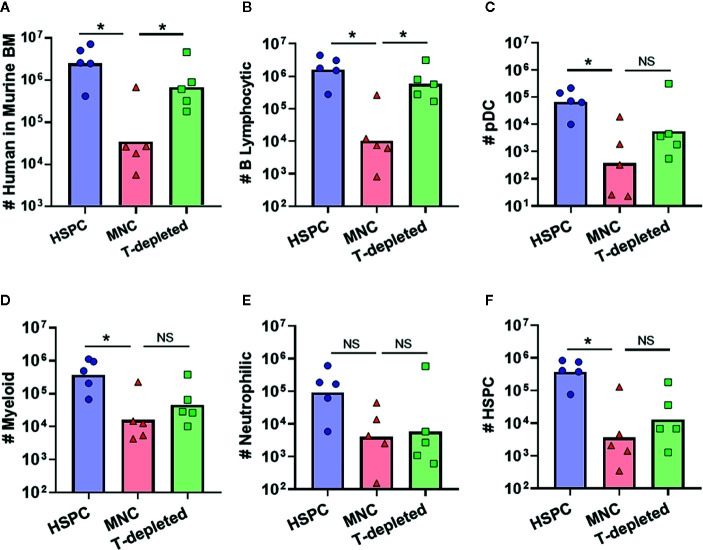
Impact of other donor-derived cells on hematopoietic output. To determine how graft composition alters hematopoietic potential and lineage commitment, transplantation of isolated BM-HSPC (blue) was compared to total bone marrow mononuclear cells (MNC, red), or T cell-depleted MNC (green). For all conditions, the grafts contained 5E^5^ HSPCs. **(A)** Plot showing total number of human cells in the bone marrow collected from one leg (femur + tibia) at 12 weeks post-transplantation. **(B–F)** Plots showing the total number of human cells of the indicated lineages for each graft condition. Symbols show results from individual mice, and bars show the geometric means. *p < 0.05.

Most clinical HSCT protocols are performed using graft tissues that contain a wide variety of immune cells, including a substantial proportion of mature T lymphocytes (29, 30). To determine how the presence of such donor-derived immune cells in graft tissue affects hematopoietic outcomes, we transplanted NBSGW mice with either isolated human BM-HSPCs, total BM mononuclear cells (MNC), or T cell-depleted MNC. Compared to transplantation of isolated BM-HSPCs, transplantation of total MNCs containing the same number of HSPCs led to approximately 2 logs lower levels of human chimerism in the murine bone marrow after 12 weeks (**Figure 7A**). However, transplantation of T cell-depleted MNCs resulted in human chimerism levels approaching those of mice that received isolated BM-HSPCs (**Figure 7A**). Since mice that showed evidence of GVHD were excluded from these analyses, this suggested that the presence of donor-derived T cells adversely affects hematopoietic output independently of GVHD pathology. Remarkably, further analysis revealed that mice receiving T-depleted MNCs showed recovery of B lymphocytic cell numbers, but that other lineages and HSPC numbers remained reduced, similar to mice that received total MNCs (**Figures 7B–F**). Thus, the presence of donor-derived T cells appeared to selectively reduce the hematopoietic output of B lineage cells, while other donor-derived cells adversely affected the production of myeloid, neutrophilic, and progenitor populations.

## Discussion

In this study, we used the NBSGW mouse strain as a model system to investigate hematopoietic output by human HSPCs. Consistent with results from prior studies that transplanted human HSPCs into immunodeficient mice bearing hypomorphic c-kit mutations (13–20), we found that a complex multi-lineage human immune compartment was generated that included B lymphocytic, neutrophilic, and multiple myeloid lineage populations. However, whereas prior studies have highlighted that Kit-mutant mice support enhanced engraftment of human myeloid cells (13–15, 17–20), our analysis revealed marked differences in the composition and duration of the human immune compartment depending on the source of the HSPCs. Of particular interest, we found that transplantation of HSPCs purified from G-CSF mobilized blood led to the generation of abundant human neutrophilic lineage cells, whereas HSPCs from the bone marrow favored B lymphocytic reconstitution, and HSPCs from the umbilical cord blood led to more durable B lymphocytic engraftment. Thus, our findings suggest that while the host environment of NBSGW mice is favorable for human myelopoiesis, it also does not overridingly skew hematopoietic output toward myeloid lineages and thus allows for distinct programs of human HSPC differentiation.

Prior human HSPC engraftment studies using irradiated immunodeficient mice with wild-type *c-kit* led to the production of a human immune compartment dominated by B cells, with comparatively low representation of myeloid lineages and little or no evidence of granulocytic lineage development (31–33). This B lymphocytic lineage bias was thought to be due to the abundance of murine IL-7, which promotes lymphopoiesis and is cross-reactive between mouse and humans (34), while murine IL-3 and GM-CSF do not appear to efficiently promote human myelopoiesis. To address this limitation, the NSG-SGM3 and MISTRG next-generation immunodeficient mouse strains were designed to support myeloid development by providing human GM-CSF and IL-3 through gene knock-in technology (8–10). While these mouse strains do support human myeloid development exceptionally well, it is not clear whether they may skew HSPCs towards myelopoiesis and thus may mask the impact on hematopoiesis of other biological features of HSPCs or of accessory cell populations. Thus, immunodeficient Kit-mutant mouse strains provide an important alternative, since the presence of hypofunctional c-kit in the endogenous murine cells and the cross-reactivity of murine SCF apparently provide a sufficient advantage to transplanted human HSPCs so that they can successfully engraft, while also supporting varied differentiation outputs.

Another important factor is that pre-transplant conditioning is not required for human hematopoietic engraftment in Kit-mutant strains, such as NBSGW. Eliminating pre-conditioning removes the influx of DAMPs, alarmins, and other inflammatory signals that could alter survival of the donor HSPCs or affect lineage differentiation and that are now known to promote the development of GVHD (3, 4, 23). Additionally, eliminating pre-conditioning leaves mesenchymal and endothelial cells undamaged, which is likely to promote hematopoietic reconstitution as it has recently been shown that HSPCs reside in specific pockets within bone marrow tissue, where they are surrounded by mesenchymal and endothelial cells that secrete cytokines important for HSPC survival and differentiation (11). Indeed, recognition of the adverse impact of pre-transplant conditioning has led to increasing interest in the use of reduced intensity conditioning or alternative protocols for clinical HSCT. For example, it has recently been shown that depleting endogenous murine HSPCs by administering an antibody-bead conjugate against CD117, allows for the survival and differentiation of human HSPCs in non-conditioned NSG mice (35, 36). With this approach now entering phase-I clinical trials, murine models that provide an HSPC-deficient host environment will likely become increasingly useful tools for further research.

A highly novel observation from this study is the efficient engraftment and abundant neutrophilic population generated by transplantation of MB-HSPCs (**Figures 4B, E**). Clinical reports have shown that patients given MB grafts tend to achieve neutrophil and platelet reconstitution faster than those receiving either BM or CB grafts (3, 4). The robust granulocytic differentiation we observed is most likely due to the prior systemic G-CSF treatment of mobilized blood donors, since G-CSF is known to induce granulopoiesis as well as mobilizing HSPCs to leave the bone marrow (25, 26). One possibility is that G-CSF treatment preferentially mobilizes HSPCs that are already committed towards granulocytic lineage differentiation. However, our analysis of the HSPC population in MB graft tissue revealed a higher frequency of CD38^−^ uncommitted progenitors than BM (**Figures 1E–H**), suggesting that the granulocytic differentiation bias of MB-HSPCs may be due to changes prior to lineage commitment. It will thus be of great interest to determine in future studies whether exposure to G-CSF alters the composition or programming of uncommitted HSPCs or instead results in selective mobilization of specific HSPC populations from the bone marrow (37). Additionally, we observed that the percentage of long-term HSCs (CD45RA^-^/CD49f^+^) was significantly lower in MB than BM (**Figure 1I**). Thus, an additional important question not addressed by our study is whether the enhanced early hematopoietic output and granulopoiesis of MB-HSPCs is accompanied by a shorter duration of engraftment in NBSGW compared to clinical transplantation protocols are typically performed using grafts that contain an abundance of lineage-committed cell types in addition to HSPCs, we also investigated the impact of such cells on hematopoietic output in our model. Prior studies have established adverse effects from the presence of T cells within graft tissues that occur even in the absence of GVHD (23, 38, 39). This may be due to low levels of IFN-*γ* production, since this cytokine has been shown to have bi-specific activity on HSPCs. Acute IFN-*γ* exposure has been shown to induce both myelopoiesis and granulopoiesis in response to an infection, but chronic IFN-*γ* exposure has been shown to cause HSPC arrest through the binding and occlusion of the HSPC pro-survival factor thrombopoietin (TPO) to its cognate receptor c-MPL (40, 41). Surprisingly, we found that removing donor-derived T cells from total MNC grafts resulted in a selective enhancement of B cell numbers, but did not significantly improve the output for other populations (**Figure 7**). This suggests that T cells may inhibit B cell engraftment, but other cells found within the MNCs may suppress the differentiation of other populations. Alternatively, T cells may override the B cell promoting effects of another population found in the MNCs. Consistent with this possibility, it has recently been shown that B lymphocytes may engage in a feed-back loop that promotes B cell development *via* a GABAergic pathway (42). Thus, a key area of future investigation will be to determine the mechanisms involved in the impact of non-HSPCs on hematopoietic output.

Lastly, it is important to acknowledge that a limitation of our study is that we did not address the functionality of the human immune lineages produced in these models. In particular, it will be important to determine whether the neutrophilic cells observed in the bone marrow develop into fully mature and functional human neutrophils, and whether these exit to the periphery. Nevertheless, this study demonstrates the potential for using NBSGW mice engrafted with human MB-HSPCs to study aspects of human granulopoiesis *in vivo*. Similarly, we show the utility of the NBSGW strain as a tool to investigate differences within HSPC populations from different human graft tissues, which remains an understudied aspect of the many variables that influence immune reconstitution following allogeneic HSCT.

## Data Availability Statement 

The raw data supporting the conclusions of this article will be made available by the authors, without undue reservation.

## Ethics Statement

The studies involving human participants were reviewed and approved by University of Wisconsin Minimal Risk Institutional Review Board. Written informed consent for participation was not required for this study in accordance with the national legislation and the institutional requirements. The animal study was reviewed and approved by University of Wisconsin Care and Use Committee.

## Author Contributions

NJH and JEG designed the study. AWH and PH procured samples. NJH, PNL, JV and SG performed the research and analyzed the data. NJH and JEG wrote the manuscript. NJH, AWH, AKS, AI, PH and JEG edited the manuscript. All authors approved the submitted version.

## Funding

NJH was supported by NIH T32 AI125231 and T32 HL07899. Additional support from NIH R21 AI116007 and R01 AI135600 (JEG) and R21 AI105841 (AWH).

## Conflict of Interest

The authors declare that the research was conducted in the absence of any commercial or financial relationships that could be construed as a potential conflict of interest.
